# Supplementary material for: Facile Microfluidic Fabrication of Biocompatible Hydrogel Microspheres in a Novel Microfluidic Device
Source: Molecules. 2022 Jun 22;27(13):4013. doi: 10.3390/molecules27134013 (PMC9268728; doi:10.3390/molecules27134013)
Supplement: Supplementary file 1 [file molecules-27-04013-s001.zip › molecules-1756633-supplementary.pdf]

molecules

MDPI

Article

Facile Microfluidic Fabrication of Biocompatible Hydrogel Microspheres in a Novel Microfluidic Device

Minjun Chen, Ruqaiya Aluunmani, Guido Bolognesi and Goran T. Vladisavljević *

[1] Department of Chemical Engineering, Loughborough University, Loughborough LE11 3TU, UK; G.Vladisavljevic@lboro.ac.uk (G.T.V.); M.Chen2@lboro.ac.uk (M.C.)

* Correspondence: G.Vladisavljevic@lboro.ac.uk (G.T.V.); Tel.: +44(0)1509-222518 (G.T.V.)

Supplementary Materials

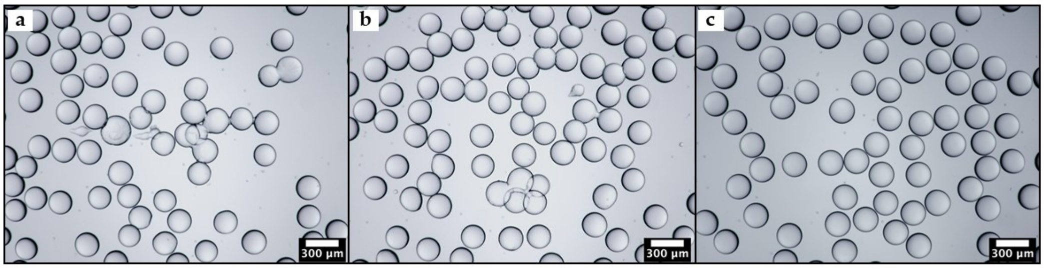

Figure S1. Optical microscopy images of generated PEGDA-99 droplets produced using different concentrations of Xiameter™ RSN-0749 in silicone oil: **(a)** 2 wt%; **(b)** 3 wt%; and **(c)** 3.5 wt%. The droplet diameter was 107 µm in all images.

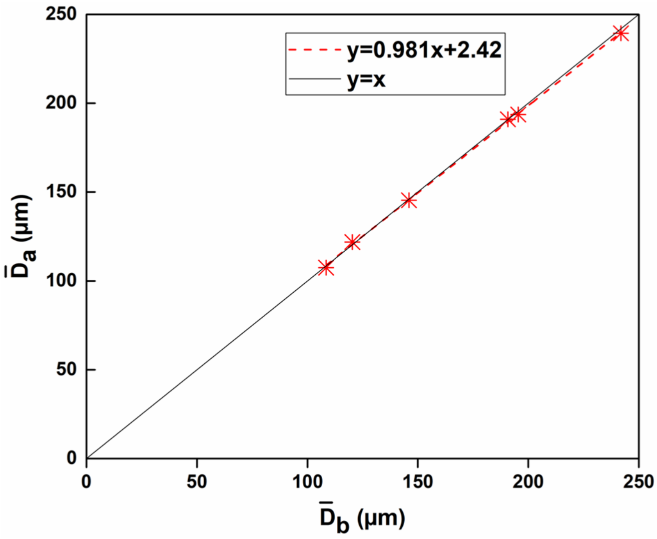

Figure S2. Comparison between the average droplet size measured using the "Oval" tool in ImageJ ($\bar{D}_a$) and ImageJ's Hough Circle Transform plugin ($\bar{D}_b$). The two methods are almost in a perfect agreement, since all the paired data points are very close to the diagonal line. Droplets are generated using different orifice sizes at the flow ratio of 6.

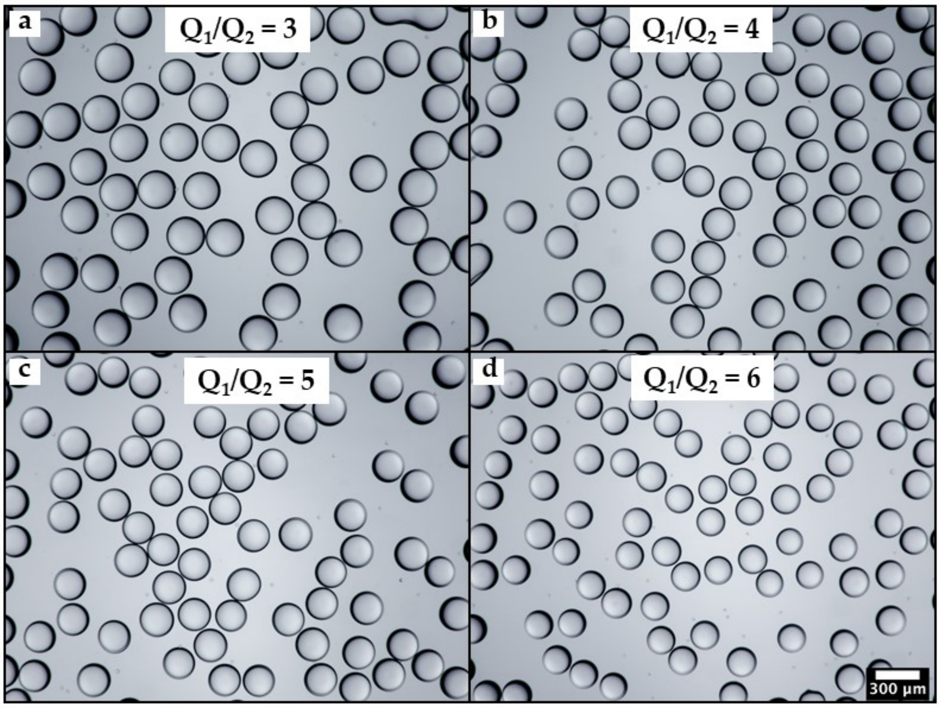

Figure S3. Optical microscopy images of particles produced at different dispersed to continuous phase flow rate ratios using collection capillary with and orifice size of D_o = 350 μm.

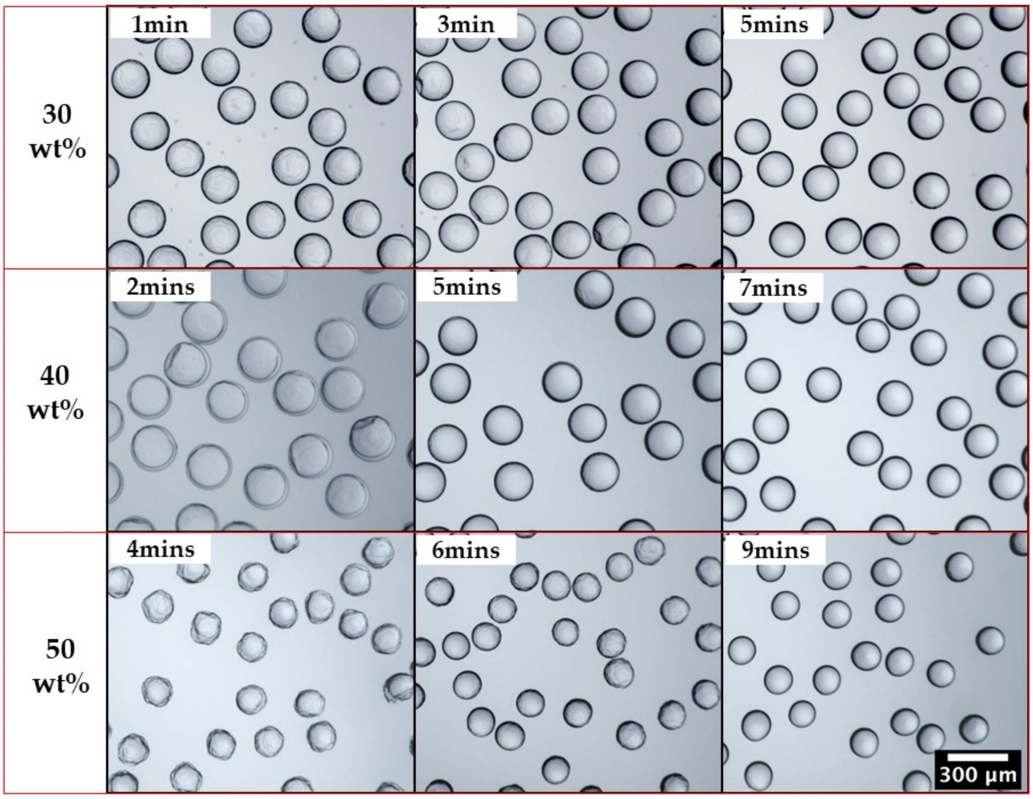

Figure S4. Images of PEGDA particles produced using the dispersed phase with different water contents and different UV light irradiation time. The same scale bar applies to all figures.
